# Microalgae-based wastewater treatment for developing economic and environmental sustainability: Current status and future prospects

**DOI:** 10.3389/fbioe.2022.904046

**Published:** 2022-09-07

**Authors:** Piroonporn Srimongkol, Papassara Sangtanoo, Pajareeya Songserm, Wannapawn Watsuntorn, Aphichart Karnchanatat

**Affiliations:** ^1^ Center of Excellence in Bioconversion and Bioseparation for Platform Chemical Production, Institute of Biotechnology and Genetic Engineering, Chulalongkorn University, Pathumwan, Bangkok, Thailand; ^2^ Panyapiwat Institute of Management Demonstration School, Pakkred, Nonthaburi, Thailand

**Keywords:** microalgae, wastewater treatment, biomolecule production, biorefineries, bioenergy

## Abstract

Over the last several decades, concerns about climate change and pollution due to human activity has gained widespread attention. Microalgae have been proposed as a suitable biological platform to reduce carbon dioxide, a major greenhouse gas, while also creating commercial sources of high-value compounds such as medicines, cosmetics, food, feed, and biofuel. Industrialization of microalgae culture and valorization is still limited by significant challenges in scaling up the production processes due to economic constraints and productivity capacities. Therefore, a boost in resource usage efficiency is required. This enhancement not only lowers manufacturing costs but also enhancing the long-term viability of microalgae-based products. Using wastewater as a nutrient source is a great way to reduce manufacturing costs. Furthermore, water scarcity is one of the most important global challenges. In recent decades, industrialization, globalization, and population growth have all impacted freshwater resources. Moreover, high amounts of organic and inorganic toxins in the water due to the disposal of waste into rivers can have severe impacts on human and animal health. Microalgae cultures are a sustainable solution to tertiary and quaternary treatments since they have the ability to digest complex contaminants. This review presents biorefineries based on microalgae from all angles, including the potential for environmental pollution remediation as well as applications for bioenergy and value-added biomolecule production. An overview of current information about microalgae-based technology and a discussion of the associated hazards and opportunities for the bioeconomy are highlighted.

## Graphical Abstract

**Figure:**
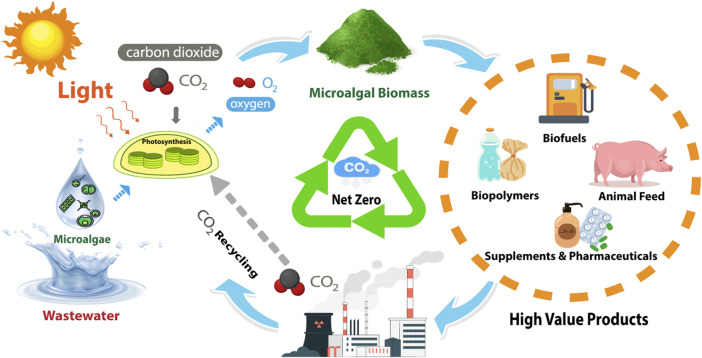
A biorefinery based on microalgae promotes a circular-green economic model.

## 1 Introduction

At present, global warming issues are escalating at an alarming degree and are becoming increasingly interconnected with others. Extreme weather events such as heat waves, droughts, and floods are becoming more frequent and intense as a result of climate change and putting human security at risk. Meanwhile, global warming exacerbates issues such as soil degradation, biodiversity loss, disease transmission, as well as water shortages. It has been speculated that these changes will have an impact on patterns of economic development, political stability, and the well-being of people. Carbon dioxide (CO_2_), a significant greenhouse gas (GHG) present in the Earth’s atmosphere that accounts for up to 60% of all greenhouse gases, has been increasing as a result of different human activities that contribute to global warming. CO_2_ emissions began to rapidly increase in the 1950s, reaching 25.23 billion metric tons of CO_2_ emission in 2000. Between 2000 and 2010, the emission of CO_2_ increased by 32%, reaching 34.81 billion metric tons in 2020. In 2020, Thailand accounted for 0.76 percent of world emissions (258 million tons of CO_2_) and was placed 24th in terms of CO_2_ emissions globally (Global Carbon Project, 2021). Moreover, Thailand is ranked by Germanwatch as one of the top ten countries at high risk of long-term climate change (Eckstein et al., 2021). Since Thailand is a developing country that depends on fossil fuel energy and has expanding metropolitan areas, the Thai government, like governments in many other countries around the world, has focused on how to reduce CO_2_ emissions by 7.6 percent per year for the next decade in order for Thailand to meet its commitment to prevent global warming from exceeding 1.5°C over pre-industrial levels. This goal, laid out in the 2015 Paris climate agreement, must be met in order to satisfy the demands of sustainable corporate practices in social and environmental responsibility, on which many nations have agreed (Tollefson, 2021).

There are two effective ways to reduce carbon dioxide emissions, including the use of alternative energy and expanding the techniques to capture and store CO_2_ to reduce emissions over the long term. Different biological, chemical, or physical processes can be used to capture and store CO_2_. Nonetheless, existing technologies have a number of technical and financial restrictions. Consequently, it is imperative that current technologies be upgraded and new ones be developed. As a comparison, biological CO_2_ fixation seems to be a more cost-effective and environmentally-friendly technology than physical or chemical methods. Biological CO_2_ fixation through photosynthesis is the process by which photosynthetic organisms absorb CO_2_, thus assisting in the control of atmospheric CO_2_ levels (Daneshvar et al., 2022). Compared to terrestrial crops, microalgae can grow faster, are more adaptable, and can fix CO_2_ at a rate that is 10–50 times greater than other land plants (Batista et al., 2015; Cuellar-Bermudez et al., 2015). Moreover, carbon sequestration using microalgae has been hailed as one of the most important and successful technologies in the world over the last few decades (Alami et al., 2021). Additionally, microalgae have the ability to survive in a wide variety of conditions without competing for food with humans and animals. Microalgae have a diverse range of functional elements, including peptides, carbohydrates, lipids, pigments, vitamins, and minerals, all of which have been shown to provide a variety of benefits. Microalgae have therefore generated widespread attention due to their potential use in various fields such as medicine, aquaculture, animal and human nutritional supplementation, agricultural production, and bioenergy.

Despite the enormous promise of microalgae in a variety of applications, their use is currently limited to laboratory settings. For a variety of reasons, industrial level applications have not been taken forward as much as they could, with the fundamental issue being the high economic costs associated with large-scale applications. The high cost of artificial medium and the poor biomass yield are two constraints connected with the generation of raw materials for diverse applications by microalgae (Pandey et al., 2019). To solve this problem, the use of effluents to culture microalgae is one of the most effective ways to emerge as a viable option to lower process costs and obtain microalgae biomass for a range of applications. Many studies reveal that microalgae can completely remove nitrogen, phosphorus, and hazardous components from various types of wastewater, resulting in biomass production, including from municipal, industrial, agro-industrial, and livestock wastewaters (Udaiyappan et al., 2017; Srinuanpan et al., 2020). Because certain microalgae species have evolved to survive in wastewater, this strategy can reduce manufacturing costs by combining wastewater treatment with the cultivation of microalgae. Thus, including microalgae-mediated CO_2_ bio-mitigation into a wastewater treatment infrastructure may be more affordable, cost-effective, and ecofriendly (Basu et al., 2014).

There have been recent advancements in the use of microalgae for environmental issues such as coupling with wastewater treatment or CO_2_ absorption. Only a limited number of studies have addressed the advantages and limitations of microalgal production in terms of green economy purposes. The composition of wastewater varies according to its source. It is critical to consider the ability of certain algae to grow in various types of contaminated wastewater. As a consequence, this review will involve wastewater from a variety of sources, such as municipal, agricultural, and industrial waste, that have been employed in algae growth experiments to create high-value bioactive chemicals in combination with wastewater treatment. The approach has the potential to incorporate CO_2_ reduction, bioenergy generation, and other high-value-added compounds produced by microalgae. The performance of several types of wastewater for microalgae culture is rigorously examined and assessed. This review also describes several microalgae potentials and future development trends for resource recovery. Furthermore, recent developments in the enhancement of CO_2_ collection by microalgae are reported in this review.

## 2 Green economy framework

Throughout the last several decades, the world economy has expanded at a dramatic pace. Extreme population expansion is a major issue, with the world’s population forecast to reach 8.5 billion in 2030, 9.7 billion in 2050, and 10.9 billion by 2100 (United Nations, 2019). As a consequence of the growing global population, large amounts of energy and resources have been consumed and pollution levels are high. The necessity of anticipating and preparing for these crises has been recognized and appreciated by many international organizations. Promoting a “green economy” is one such plan. The green economy concept was developed during the 2012 United Nations Conference on Sustainable Development in Rio de Janeiro, and it is based on the idea that environmental protection helps both the economy and society. The goal of this concept is to empower farmers and manufacturers to create more environmentally friendly production and consumption systems based on reuse and recycling for sustainable development (Loiseau et al., 2016).

## 3 Wastewater integrated algae-biorefinery for high-value compounds production

Because of the efficiency of being a bio-refinery of algae, interest in wastewater integrated algal-biorefinery has recently attracted considerable attention. Many previous studies indicate that several wastewaters (e.g., domestic, agricultural, and industrial wastewater) are rich in appropriate nutrients which could serve as inexpensive alternative raw nutrients source to cultivate microalgae using CO_2_ from atmospheric and flue gases (Wollmann et al., 2019; Chew et al., 2021; Li et al., 2022). The considerable advantage of wastewater integrated microalgae-based biorefinery is that it both solves environmental problems and also produces biofuel as well as other value-added compounds such as pigments, microelements, omega fatty acids, antioxidants, and animal feed (Kadir et al., 2018; Xiaogang et al., 2020; Chew et al., 2021).

**FIGURE 1:**
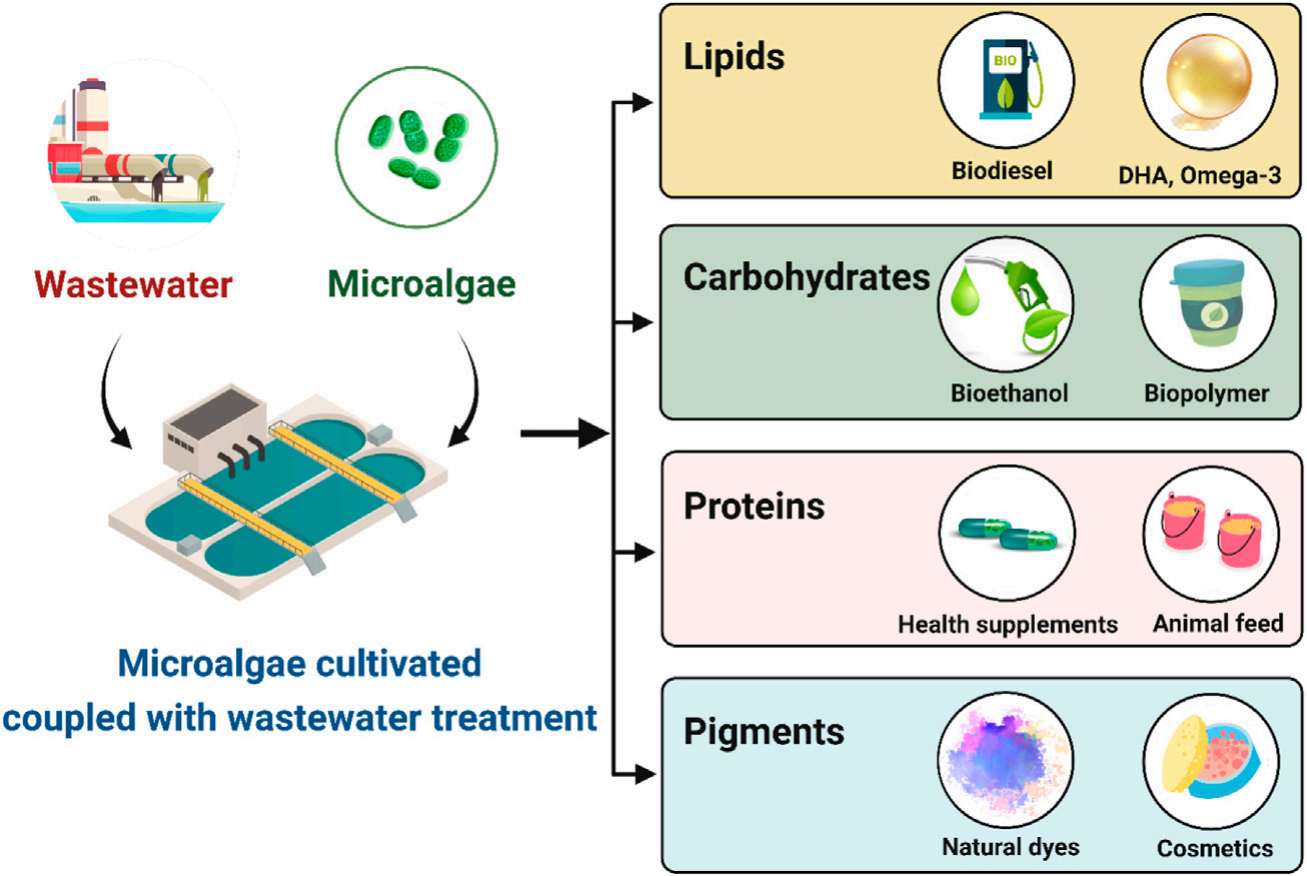
Wastewater integrated algal-biorefinery for biofuel and other value-added compound productions (created with BioRender.com).

The importance of microalgae as a rich source of novel bioactive compounds is quickly becoming recognized, as evidenced by the number of research studies on the topic. A significant number of vital compounds such as fatty acids, pigments, and other biochemicals are found in the composition of microalgae, resulting in microalgae becoming increasingly important (Bhattacharya and Goswami, 2020). A lot of biomolecules produced by microalgae have a diverse range of applications. Many different types of molecules can be utilized for a variety of different purposes, ranging from the use of lipids, proteins, and carbohydrates in food and nutraceutical applications to the use of pigments and sterols in cosmetic and pharmaceutical applications. To facilitate this review, the compounds isolated from microalgae are illustrated in Figure 1 with respect to four different domains of nutrition: carbohydrate, protein, lipid and pigment. According to global microalgae market data, microalgae pigments and lipid are two of the most prominent microalgae products on the market. The green alga *Dunaliella salina*, which thrives in open ponds with high salinity and light, is the source of the pigment β-carotene, which was created in massive quantities in 2010 (Enzing et al., 2014). The freshwater green alga *Haematococcus pluvialis* produces the commercially important pigment astaxanthin (Shah et al., 2016). Astaxanthin has a larger market value than β-carotene, despite its lower output. However, astaxanthin’s commercialization has been slow due to high production costs and the lack of a recognized market for human usage (Borowitzka, 2013). In the last decade, microalgae have become a third-generation biofuel feedstock due to their high triglyceride (TAG). However, the majority of energy is derived from nonrenewable fossil fuels such as coal, petroleum oil, and natural gas. The demand for energy is always increasing because of rapid population growth and industrialization. Consequently, the search for alternate energy sources has piqued the public’s interest. The potential use of algae as a source of renewable energy has inspired considerable interest due to a number of key advantages, including a rapid growth rate, high lipid content, and the capacity to grow without the use of arable land. Furthermore, algae can efficiently take CO_2_ during photosynthesis and create polysaccharides and triacylglycerol (TAG) (Wijffels and Barbosa, 2010). These compounds are the starting materials for the synthesis of bioethanol and biodiesel, both of which may be used in existing engines without major changes. Details of these products are discussed in the next topic.

### 3.1 Biofuel production

Microalgae have a capacity as a producer comparable to that of a land plant, which has been critical to human survival for a long time in human history in terms of significant sources of food, medicine, building materials, and energy supply. Diverse photosynthetic unicellular microalgae are emerging as novel sources of renewable energy that can fulfill the demands of the human activities. Microalgae lipids can be utilized as a raw material for biodiesel synthesis, and remaining biomass rich in carbohydrates can be used to produce bioethanol or biogas. Furthermore, using various thermochemical conversion processes, the whole biomass may be turned straight into crude bio-oil (Wang et al., 2022). Microalgae typically accumulate lipids between 20–50% of their dry weight. Some species can be as high as 80% under certain condition (Chisti, 2007). These neutral lipids, mainly in the form of triacylglycerols (TAGs) (up to 90–95%), which can be transformed to fatty acid methyl esters (FAMEs) and converted biodiesel (MacDougall et al., 2011). Algae have advantages over first generation biofuels made from sugar, starch, and vegetable oil because of their high growth rates and productivity, ability to grow on non-arable land using wastewater, ability to use water contaminants and CO_2_, and ability to produce a variety of high-value biological compounds (Cheng and Luo, 2022). In 2016, 135 billion gallons of biofuels (biodiesel and ethanol) were produced globally, accounting for 4% of total transportation fuel use. Biodiesel and ethanol are alternatives for petroleum diesel and gasoline, respectively, and have many advantages over petroleum, including lower CO_2_ emissions, lower manufacturing costs, and more sustainability (Kushwaha et al., 2022). In the global scenario, unpredictable energy prices are projected to persist due to rising demand from fast growing global economies, limited crude oil reserves, and political upheaval in crude oil producing countries, which can create significant supply disruption, speculation, and so on. This insecurity has the potential to affect the entire economy of the country and the world. As a result, liquid biofuels are now seen as an unavoidable development in the field of renewable energy. Table 1 shows several studies of microalgal growth coupled with wastewater treatment for biodiesel, bioethanol, biogas, biohydrogen and other valuable compounds production.

**TABLE 1:** Several studies of microalgae growth coupled with wastewater treatment for biofuel and other value-added compounds production.

Microalgae	Sources of wastewater	Process	Bioproduct	References
Microalgae consortium	85–90% carpet industry effluents with 10–15% municipal sewage	Cultured in Erlenmeyer flasks	63.9% biodiesel of algal oil	Chinnasamy et al. (2010)
*Chlorella zofingiensis*	Piggery wastewater	Semi-continuous feeding operation in the tubular bubble column photobioreactors under outdoor conditions	9.19 g biodiesel/100 g dry weight	Zhu et al. (2013)
*Scenedesmus obliquus, Micractinium pusillum, Dictyosphaerium pulchellum and Coelastrum* sp	Municipal wastewater	Cultivated in High-Rate Algal Pond (HRAP)	70.9% yield of biodiesel	Doma et al. (2016)
Microalgae	Wastewater-grown microalgae	Fermentation by *Clostridium saccharoperbutylacetonicum* N1-4 using wastewater algae biomass pretreated with xylanase and cellulase enzymes	9.74 g L^−1^ of total acetone, butanol, and ethanol (ABE)	Ellis et al. (2012)
*Nannochloropsis gaditana *	Municipal wastewater	Microalgae biomass production and fermentation	94.3 mg ethanol/g biomass	Onay, (2018)
*C. vulgaris*	Wastewater treatment effluent from tilapia culture pond	Microalgae biomass production and fermentation	33.213 g ethanol L^−1^	Bhuyar et al. (2021)
*S. obliquus*	Urban wastewater	Microalgae biomass production and dark fermentation using *Enterobacter aerogenes*	56.8 ml H_2_ g^−1^ VS.	Batista et al. (2015)
*S. obliquus*	Artificial wastewater	Sulfur deprivation and two light quality priors to anaerobic condition	128 ml H_2_ L^−1^ (productivity 204.8 ml H_2_ L^−1^ d^−1^)	Ruiz-Marin et al. (2020)
*C. vulgaris* and mixed culture of native algae	Synthetic and wastewater media	Cultured in synthetic medium, wastewater (sterilized and non-sterilized) and digestate from anaerobic digestion of pulp and paper biosludge (sterilized and non-sterilized)	154–252 L CH_4_ kg^−1^ (depending on culture media)	Kinnunen and Rintala, (2016)
*Chlorella* sp. and *Scenedesmus* sp.	Domestic sewage	High-rate algal ponds (HRAP) for post-treating Up flow anaerobic sludge blanket (UASB) reactors’ effluent and anaerobic co-digestion with microalgae	156–211 NL CH_4_ kg_vs_^−1^	Vassalle et al. (2020)
*H. pluvialis*	Primary-treated sewage (PTS), Primary-treated piggery wastewater (PTP)	Cultured in wastewater with serial dilution	5.1 and 5.9% of the total biomass of the PTS and PTP	Kang et al. (2006)
*Nostoc* sp., *Arthrospira platensis* and *Porphyridium purpureum*	Industrial wastewater	Cultured in Erlenmeyer flasks	179 mg phycobiliproteins/g dry weight	Arashiro et al. (2020)
*C. vulgaris*,*S. obliquus*	Aquaculture effluents	Cultured in Erlenmeyer flasks sealed with hydrophobic cotton and agitated by an air flow	31% proteins, 6% lipids and 39% carbohydrates of *C. vulgaris* biomass, 35% proteins, 8% lipids, and 30% carbohydrates of *S. obliquus* biomass	Viegas et al. (2021)

### 3.2 Biodiesel

The production of biodiesel from microalgae comprises two different steps: 1) lipid extraction from microalgal cells and 2) transesterification of lipids using alcohol and a catalyst (Mondal et al., 2017). In a preliminary study of biomass production and ammonium removal in *Synechococcus* sp. VDW (accession number MH393765) isolated from natural seawater in Thailand (Tinpranee et al., 2018), we discovered that at optimum conditions (initial pH 7.4, inoculum size of 0.17 (OD_730_), and ammonium concentration of 10.5 mg L^−1^), maximum ammonium removal and biomass productivity were 95% and 34 mg L^−1d−1^, respectively. Furthermore, fatty acid methyl ester (FAME) analysis revealed that the major fatty acids were palmitic acid (C16:0), linoleic acid (C18:2 n6 cis), palmitoleic acid (C16:1), and oleic acid (C18:1 n9 cis), accounting for more than 80% of total fatty acids, indicating that this strain has potential for simultaneous water treatment and biomass production for biofuel feedstock (Srimongkol et al., 2019b). A review study by Pancha et al. (2019) showed various microalgae lipid content cultivated in various wastewaters ranging from 18–79% w/w of biomass. Meanwhile, Chinnasamy et al. (2010) found that >96% nutrient in treated wastewater containing 85–90% carpet industry effluents with 10–15% municipal sewage could be removed by a range of native algal isolates. Biomass production potential and lipid content were ∼9.2–17.8 tons ha^−1^ year^−1^ and 6.82%, respectively. In addition, around 63.9% of algal oil could be converted into biodiesel. In addition, Zhu et al. (2013) reported the FAME yield of *C. zofingiensis* in piggery wastewater for outdoor simultaneous wastewater treatment was 9.19% of dw. Doma et al. (2016) showed the oil content of biomass collected from High-Rate Algal Pond (HRAP) constructed to treat municipal wastewater was 5% and the biodiesel yield was 70.9%.

### 3.3 Bioethanol

Microalgae can produce and accumulate an abundance of carbohydrates that are useful for bioethanol production (Maia et al., 2020). Generally, wastewater-grown microalgae require pretreatment to hydrolyze the complex sugars into simple and readily metabolizable carbon source by fermentative microorganisms (Pancha et al., 2019). Ellis et al. (2012) showed that 9.74 g L^−1^ acetone-butanol-ethanol (ABE) was produced by *Clostridium* spp. Using wastewater algae biomass as a carbon source. Onay (2018) demonstrated that bioethanol yields of *Nannochloropsis gaditana* in various concentrations of municipal wastewaters (0, 30, 60, and 100%) ranging from 70.3 ± 2.4 mg g biomass^−1^ to 94.3 ± 5.5 mg g biomass^−1^ and 30% of wastewater showed the highest bioethanol yield (94.3 ± 5.5 mg g^−1^ biomass). A recent study by Bhuyar et al., 2021 showed that *C. vulgaris* cultivation in wastewater effluent from tilapia culture pond produced biomass of 0.376 ± 94.21 mg L^−1^ after cultivation and produced the highest ethanol concentration of 33.213 g L^−1^ after 96 h of fermentation.

### 3.4 Biohydrogen

Biohydrogen production from microalgae can take place through in different routes but generally involves fermentation biohydrogen production (e.g., dark fermentation biohydrogen production, photo fermentation biohydrogen production, and photo-dark combined fermentation biohydrogen production) and photosynthesis biohydrogen production (e.g., direct biological photolysis biohydrogen production, indirect biological photolysis biohydrogen production) (Wang et al., 2021). Batista et al. (2015) reported that *S. obliquus* can grow in urban wastewater and then the biomass can be converted into biohydrogen through dark fermentation by *Enterobacter aerogenes* producing 56.8 ml H_2_ gvs^−1^. Ruiz-Marin et al. (2020) demonstrated that microalgae *C. vulgaris* and *S. obliquus* immobilized cells grown in urban wastewater can produce biohydrogen in sulfur deprivation with a maximum hydrogen production of 128 ml H_2_ L^−1^ (productivity 204.8 ml H_2_ L^−1^ day^−1^) and 60.4 ml H_2_ L^−1^ (productivity 39.18 ml H_2_ L^−1^ day^−1^) for *S. obliquus* and *C. vulgaris*, respectively.

### 3.5 Biogas

Biogas is the end product of anaerobic digestion, which produces gas composed of 50–70% of methane, 30–45% of carbon dioxide, < 2% of hydrogen, and <3.5% of hydrogen sulfide (Vanegas and Bartlett, 2013; Milledge et al., 2019). Generally, anaerobic digestion is conducted by two processes: 1) simple sugar is fermented by fermentative bacteria and converted into alcohols through anaerobic digest and 2) metanogenic microorganisms use these compounds and synthesize biomethane (Danquah et al., 2011). Choudhary et al. (2016) revealed that native consortia PA6 has good nutrient removal ability from rural wastewaters with a theoretical methane potential of up to 0.79 m^3^ kg^−1^ VS. Kinnunen and Rintala (2016) showed that the biomethane potential of *C. vulgaris* and mixed culture of native algae species (dominating by *Scenedesmus* sp.) varied between 154 and 252 L CH_4_ kg^−1^ VS. depending on culture media including synthetic medium, wastewater (sterilized and non-sterilized), and digestate from anaerobic digestion of pulp and paper biosludge (sterilized and non-sterilized). A recent study by Vassalle et al. (2020) evaluated sewage treatment efficiency and biogas production of an up flow anaerobic sludge blanket (UASB) reactor followed by a high-rate algal ponds (HRAP) during a 1 year at demonstration–scale. Their result indicates that 65% COD and 61% N-NH_4_ were overall removed from the system. In addition, methane yield increased by 25% after anaerobic co-digestion with microalgae (156–211 NL CH_4_ kgVS^−1^).

### 3.6 Other valuable compounds

Despite intensive studies and several scientific initiatives, commercialization of microalgal biofuels has not yet been achieved. Since it is not economically feasible to devote resources to the production of a single microalgal product, researchers have found that combining the development of numerous products from a single batch of biomass is more efficient. Therefore, to produce biofuels, microalgal biomass can also be used for other purposes to produce other value-added compounds (Xiaogang et al., 2020). Several bioactive compounds have been discovered and purified from marine microalgae, including polysaccharides, pigments, and fatty acids (Ryckebosch et al., 2014). Some of these metabolites have demonstrated biological activities, including anti-oxidant, anti-inflammatory, anti-cancer, and anti-viral properties (Table 2). Among them, the most abundant components are proteins that play an important role in the structure and metabolism of microalgal cells. They are an essential component of the membrane and light-harvesting complex, which contains a large number of catalytic enzymes that are involved in the photosynthesis process. In terms of quantity, several species of microalgae have been reported to contain extremely high concentrations of protein; on a dry weight basis, these concentrations can range from 42% to over 70% in certain cyanobacteria and up to 58% in *C. vulgaris*, according to the literature (Hachicha et al., 2022). Microalgae are high in nutritional value because they contain all of the essential amino acids that mammals are unable to produce. Recently, peptides derived from the enzymatic hydrolysis of various dietary proteins have been shown to have a wide range of bioactivities, and there have been numerous papers on anti-oxidants and anti-cancer properties (Sheih et al., 2010; Ko et al., 2012; Zhu et al., 2017; Suttisuwan et al., 2019a; Suttisuwan et al., 2019b; Moaveni et al., 2022). For example, Angiotensin-converting enzyme (ACE) inhibitory and antioxidant properties from microalgae *C. vulgaris* were reported by Sheih et al., 2010) and they then discovered that the peptide fraction isolated from pepsin hydrolyzed algae protein waste had strong dose-dependent antiproliferation and induced post-G1 cell cycle arrest in human gastric cancer cell lines AGS. Carbohydrates, which include monosaccharides, oligosaccharides, and polysaccharides, perform structural and metabolic activities. In particular, an exopolysaccharide derived from microalgae secretions has attracted considerable attention due to its ease of extraction and isolation from the medium, which saves considerable time and energy. Exopolysaccharides are a highly valuable class of chemicals which have been demonstrated to possess immunomodulatory, anticoagulant, antimutagenic, antibacterial, radioprotective, anticancer, and anti-inflammatory bioactivities (Yim et al., 2003; Kanekiyo et al., 2005; Ngatu et al., 2012; Bafana, 2013). Glycosyllipids and triacylglycerols are two examples of microalgal lipids that can be classified as either polar or neutral. Cell membranes and organelles mostly contain polar lipids, while glycerol and unsaturated fatty acids (UFAs) are energy-storing molecules (Lupette and Benning, 2020). The amount of lipids in microalgae is affected by the type of microalgae, the amount of light, the growing environment, and the temperature. Despite these differences, microalgae are a significant source of polyunsaturated fatty acids (PUFAs), including omega-3 fatty acids, docosahexaenoic (DHA), and eicosapentaenoic (EPA). These UFAs have also been shown to have antioxidant capabilities, to reduce hypertension and to have immune-regulating qualities (Zhou et al., 2022). As a result, a number of microalgae, such as *Schizochytrium* (for DHA), *Nannochloropsis* (for EPA), *Isochrysis*, *Nannochloropsis*, *Phaeodactylum*, *Pavlova*. and *Thalassiosira* (for omega-3), have potential as fish oil substitutes for vegetarians, vegans, and those who dislike to the taste of fish. Furthermore, pigments found in microalgae such as astaxanthin, β-carotene, phycocyanin, lutein, and violaxanthin have anti-oxidant, anti-cancer, and anti-inflammatory properties (Dufosse et al., 2005; Talero et al., 2015; Chen et al., 2016; Khoo et al., 2019; Prabakaran et al., 2020). Kang et al. (2006) reported that astaxanthin content accounted for about 5.1 and 5.9% of the total biomass of *H. pluvialis* cultivation in primary-treated sewage (PTS) and primary-treated piggery wastewater (PTP), respectively.

**TABLE 2:** Nutrient compositions in different microalgae species and their health benefits.

Microalgae	Active compounds	Health benefits	References
Carbohydrate			
Chlamydomonas reinhardtii	Ara, Rha, Rib, Xyl, Gal, Glc	Antioxidant properties	Bafana, (2013)
Gyrodinium impudicum	Gal	Anti-viral activity, immunostimulatory	Yim et al. (2003)
Nostoc flagelliforme	Glu, Gal, GlcA Xyl, Man	Anti-viral activity, antithrombin activity	Kanekiyo et al. (2005)
Aphanothece sacrum	Glc, Fuc, GalA, Rha, GlcA, Gal, Man, Xyl	Anti-inflammatory, anti-allergic, adsorption of metal ions, liquid crystallization	Ngatu et al. (2012)
Protein			
Schizochytrium limacinum	Short peptide with molecular weight about 5–10 kDa	Antioxidant properties	Moaveni et al. (2022)
Synechococcus sp. VDW	Short peptide with molecular weight <3 kDa	Antioxidant, anti-inflammatory and anti-colon cancer (SW 620)	Suttisuwan et al. (2019a); Suttisuwan et al. (2019b)
C. vulgaris	Short peptide with molecular weight <1.3 kDa	Antioxidant, antiproliferation and induced a post-G1 cell cycle arrest of human gastric cancer cell lines	Sheih et al. (2010)
C. vulgaris	Di- and tri-peptides	Anti-diabetes (type 2)	Zhu et al. (2017)
C. ellipsoidea	Short peptide with molecular weight 467.2 Da	Reduces blood pressure levels	Ko et al. (2012)
Lipid			
Nannochloropsis, Schizochytrium	EPA and DHA	Protection of neurons	Lopes et al. (2017)
Phaeodactylum tricornutum	EPA	Antibacterial activity (Staphylococcus aureus)	Desbois et al. (2009)
Isochrysis, Nannochloropsis, Phaeodactylum, Pavlova and Thalassiosira	Omega-3	Fetal neurodevelopment, anti-inflammatory, antibiotic, antiproliferative, anti-arrythmic, anti-atherosclerotic, and anti-thrombotic properties	Ryckebosch et al. (2014)
S. intermedius	Fatty acid methyl esters	Antibacterial activity (Escherichia coli and Pseudomonas aeruginosa	Davoodbasha et al. (2018)
Pigment			
*H. pluvialis*	Astaxanthin	Antioxidant activity, anticancer properties and the ability to prevent diseases	Khoo et al. (2019)
Dunaliella salina	β-carotene	Preventing night blindness and liver fibrosis and improving the immune system	Dufoss ´e et al. (2005)
*Spirulina platensis*	phycocyanin	Anticancer, anti-diabetic and anti-inflammatory	Prabakaran et al. (2020)
*C. sorokiniana*	Lutein	Prevent some types of cancer and cardiovascular disease	Chen et al. (2016)
*Dunaliella tertiolecta, C. ellipsoidea*	Violaxanthin	Anti-inflammatory, anti-cancer	Talero et al. (2015)

## 4 Wastewater in an alternative growth media

Microalgal-based wastewater treatment is possible by using wastewater as a source of nutrients for microalgae growth, which promotes the concept of a circular economy and increases the sustainability of the process. In recent years, many types of wastewater have been employed to develop algal biomass for phytoremediation purposes. Wastewater is classified into different categories based on its source: municipal wastewater (produced by rural and urban households), agricultural wastewater (produced by crop cultivation, livestock breeding, agricultural product processing, and so on), and industrial wastewater (produced by various industries), as discussed in the subsection below. Table 3 summarizes the wastewater supply, nutrient removal potential, eventual biomass concentration, growing system, and production rate for each study.

**TABLE 3:** Role of microalgae in different types of wastewaters in terms of nutrient removal efficiency, incubation time, and microalgae production.

Wastewater source	Microalgae	Cultivation system	Nutrient removal efficiency (%)	Incubation time (d)	Micro algae production	References
Municipal wastewater	*Dunaliella salina*	2.5-L photobioreactors	NO_3_^−^ 88, NH_4_-N 70, TP 47.5	6	169.5 mg L^−1^ (VSS)	Liu and Yildiz, (2018)
Pretreated municipal wastewater	*S. obliquus*	continuous culture	TN:99.8 TP 83.1	9	-0.58 g L^−1^	Han et al., 2021
Primary settling tank	Mixed indigenous microalgae	Flasks	TN:99.8 TP 97.6	25	0.62 g L^−1^	AlMomani and Örmeci, (2016)
Secondary settling tank	Mixed indigenous microalgae	Flasks	TN:63.2, TP 70, NH_4_-N 63.2, COD 64.9TN:67.3 TP 30.8, NH_4_-N 67.5, COD 70.3TN:80.8, TP 50, NH_4_-N 71.1, COD 69.3TN 98 TP 25	9	1.03 g L^−1^	Aketo et al. (2020)
Primary effluent	Mixed indigenous microalgae	Photobioreactor	NH_4_-N 81.16, TP 85.29, COD 62.3	4	125 mg L^−1^ d^−1^	Lv et al. (2018)
Secondary effluent	*Parachlorella kessleri*	Flasks	TN 78.3, TP > 97.7, COD 88.8	15	1,182.5 mg L^−1^	Chen et al. (2020)
Centrate	*C. vulgaris*	Flasks	TN 85, TP 66, COD 81	10	0.29 ± 0.01 g L^−1^ d^−1^1.23 g L^−1^	Choi, (2016)
Dehydration of sludge	*C. sorokiniana*	Batch cultivation	NH_4_-N 98–100	18	0.087 g L^−1^ d^−1^	Srimongkol et al. (2019a)
Agricultural wastewater	*C. vulgaris*	Flasks	NH_4_-N > 99.99, PO₄ ¯³ > 97	14	0.0204 g L^−1^ d^−1^	Wu et al. (2017)
Undiluted cattle farm wastewater	*Synechococcus* sp VDW	Flasks	NH_4_-N-84, COD >60TN 86, 85% Reactive PO₄^−3^ 85, COD 48	7 25	0.4403 g L^−1^ d^−1^	Hariz et al. (2019)
Swine wastewater	*S. acuminatus*					
Digested dairy wastewater	*Chlorella* sp					
Brackish shrimp aquaculture wastewater	*Scenedesmus* sp. *and Chlorella* sp					
Industrial wastewater						
Pulp and paper mill biosludge digestate						
Textile wastewater, Palm oil mill effluent						

### 4.1 Composition of wastewater for microalgae cultivation

For more than 30 years, environmental concerns regarding biological and chemical water pollution have been a major focus for society, business, and government (Crini and Lichtfouse, 2019). Vast amounts of wastewater are produced which can contain harmful contaminants. Wastewater often contains substantial amounts of organic and inorganic nutrients, which generate ecological imbalances owing to their high biological oxygen demand (BOD) and chemical oxygen demand (COD). Excess nutrients, particularly nitrogen (N) and phosphorus (P), create water eutrophication, which is one of the world’s most difficult environmental concerns (Yang et al., 2008). This phenomenon causes environmental concerns such as solid waste and by-product generation, undesirable product emissions into the air, excessive growth of undesirable microbes endangering aquatic life forms, and groundwater contamination, which contribute to widespread health-related problems in areas near the discharge range (Amenorfenyo et al., 2019). It is vital to treat wastewater to reduce environmental contaminants (Rasoul-Amini et al., 2014). Wastewater treatment provides necessary protection for the sustainability of urban environment because it is a key part of global water circulation. The major objective of wastewater treatment is to markedly remove contaminating that are implanted in the water such as carbonaceous (organic; predominantly determined as biological oxygen demand (BOD)) materials, nitrogen (N), and phosphorus (P) compounds prior to being discharged into receiving systems (Grady et al., 2011; Peter et al., 2021).

Using microalgae in wastewater treatment is a sustainable option that has been widely studied for over 50 years in terms of microalgal production of useful chemical compounds, such as biofuels, as well as wastewater treatment, because it can efficiently convert carbon dioxide (CO_2_) into biofuel products and chemical substances without generating pollution and can lead to a reduction in greenhouse gas emissions. Furthermore, these procedures outweigh the disadvantages of traditional wastewater treatment, such as high operational costs and the generation of secondary waste from chemical operations (Rasoul-Amini et al., 2014; Srimongkol et al., 2019a; Aketo et al., 2020; Chai et al., 2021). It is critical to treat all forms of wastewaters in order to decrease pollutants in the environment (Rasoul-Amini et al., 2014). Wastewater composition can be greatly influenced by different wastewater generating methods and disposal systems (Bhatia et al., 2021). The composition of wastewater has a significant impact on the development of microalgae, the rate of pollutant clearance, and the creation of various intracellular compounds (carbohydrate, protein, and lipid). The carbon source, organic or inorganic carbon, macronutrients, nitrogen, phosphorus, micronutrients, vitamins, and trace elements in wastewater all have an effect on the capacity of microalgae to remove pollutants and thrive (Ahmad et al., 2022; You et al., 2022). The wastewater widely utilized for microalgae production documented in the literature may be classified according to its source, which includes municipal, agricultural, and industrial wastewater (Chiu et al., 2008; Liu and Hong, 2021).

### 4.2 Municipal wastewater

Municipal wastewater or domestic wastewater is defined as wastewater discharged from houses, kitchens, bathrooms, and laundry rooms (REF). In comparison to several types of wastewater, municipal wastewater composes of lower levels of N (15–90 mg L^−1^), P (5–20 mg L^−1^) and typically has a low level of COD concentration (less than 300 mg L^−1^) (Scott et al., 2012; You et al., 2022) and is often suitable for microalgae-based wastewater processes. Municipal wastewaters generally have a low COD concentration (less than 300 mg L^−1^) which why municipal wastewater has been the more commonly used and studied in recent decades (Li et al., 2008). There are four categories of municipal wastewater used for cultivation of microalgae, including raw sewage, which is municipal wastewater prior primary settling, primary sewage, which is wastewater after primary settling, secondary sewage, which is wastewater after treating with activated sludge in the aeration tank, and centrate, which is the by-product of sludge dewatering containing high amounts of nutrients (Li et al., 2008; Liu and Hong, 2021; You et al., 2022).

The growth and purification capabilities of *C. vulgaris*, *Neochloris oleoabundans*, and the mixed microalgal in primary sewage, secondary sewage, and centrate were investigated by AlMomani and Örmeci (2016). The results demonstrated that their growth rates differed in different types of wastewater, and the mixed indigenous microalgae showed better wastewater purification capability than *C. vulgaris*, *N. oleoabundans*. Moreover, Han et al. (2021) demonstrated that *S. obliquus* showed a vital role in removing nitrogen and phosphorus from the wastewater from primary and secondary settling tanks. The results showed that total nitrogen (TN) from primary and settling tank wastewater were 99.8 and 98.9%, respectively. Meanwhile, total phosphorus (TP) from primary and settling tank wastewater were 83.1 and 97.6%, respectively. Moreover, the total lipid yields of *S. obliquus* for 10 days cultivation in wastewater from the primary and secondary settling tanks were 0.38 and 0.33 g L^−1^, respectively which were higher than those found in the other literature. In addition, to improve the removal efficiency of nutrients in municipal wastewater using micro algae, many studies found that adding high concentration of CO_2_ (5–15%) can induce the nutrient removal and improve growth and lipid production by microalgae (Li et al., 2008; Lima et al., 2020; Liu and Hong, 2021). The nutrient composition in different municipal wastewaters should be considered as an important factor for microalgae treatment. The ratio and the nutrients concentration should be balanced by mixing different categories of wastewater to meet wastewater discharge standards. The influence of emerging pollutants on the performance of microalgae treatment should also be considered (You et al., 2022).

### 4.3 Agricultural wastewater

Agricultural wastewater is wastewater discharged from the process of crop cultivation, livestock breeding, and agricultural products processing, including farmland drainage wastewater and animal manure wastewater, (Liu and Hong, 2021; You et al., 2022). Several studies have presented the benefits of agricultural product processing wastewater as a medium for microalgae cultivation, such as potato processing wastewater, palm oil mill effluent, starch processing wastewater, and swine wastewater (You et al., 2022). Agricultural wastewaters, especially animal manure wastewaters, have high nutrient concentrations and so are great sources for nutrient recovery (Li et al., 2019). Agricultural wastewaters from animal manure wastewater have high nutrient concentrations, high turbidity, and high insoluble organic compound concentrations, and there are very limited algae species used in the animal wastewater treatment (Li et al., 2019; Liu and Hong, 2021). For example, piggery wastewater generally has N/P ratio of 12–17, a total nitrogen level of 800–2300 mg L^−1^, and total phosphorus levels of 50–230 mg L^−1^ (Beuckels, et al., 2015). However, contaminants with high turbidity can block light and reduce photosynthetic efficiency. Meanwhile, high amounts of ammonia nitrogen concentration can impede the growth of microalgae involving the electron transfer of photosystem II, making it unsuitable for microalgae cultivation. In addition, there are very limited algae strains used in the animal wastewater treatment (Zhou et al., 2012). Consequently, Therefore, agriculture wastewater is commonly diluted before algal-based treatments in order to reduce the turbidity and nutrient concentration. From Chen et al. (2020), the TN, TP and COD removal efficacies for *C. sorokiniana* strain AK-1 were 78.3 ± 1.4%, >97.7% and 88.8 ± 0.9%, respectively after 15 days of cultivation in 10% diluted swine wastewater augmented with BG11 medium.

### 4.4 Industrial wastewater

Industrial wastewater includes pulp and paper industry effluent, petroleum industrial wastewater, sugar mill effluent, coal-fired metal-contaminated wastewater, pharmaceutical industry wastewater, textile dye industry effluent, palm oil mill effluent (POME), electroplating industry wastewater, and agricultural machinery manufacturing industry wastewater (Wang et al., 2016; Udaiyappan et al., 2017). Wastewater generated from various industrial sections are composed of many types of contaminants such as heavy metals, antibiotics, oil and grease, and some other chemicals (Udaiyappan et al., 2017; Goswami et al., 2021; Liu and Hong, 2021). For example, Thailand is a major manufacturer and exporter of palm oil products. POME is wastewater generated from palm oil industry and is a known contaminant discharged into rivers in Southeast Asia. The concentrations of TN, NH_3_-N, BOD, and COD in POME are in the ranges of 180–1,400 mg L^−1^, 4–80 mg L^−1^, 10,250–43,750 mg L^−1^, and 15,000–100,000 mg L^−1^, respectively, with pH values in the range of 3.5–5.2 (Udaiyappan et al., 2017). Similarly, food processing industries which belonged to the industry section also contain high levels of TN, TP, BOD, COD, BOD, TN, and TP (Goswami et al., 2021).

Due to the aforementioned reasons, industrial wastewater is not appropriate for microalgae-based treatment based on the characteristics and properties of industrial wastewater reported by many previous studies. Only specific species of microalgae could be used to treat toxic heavy metals in wastewater through absorption and adsorption (Li et al., 2019; You et al., 2022). Microalgae, namely *Cholorella* and *Scenedesmus* have been proven as an effective species for treating olive oil and industry wastewater (Tao et al., 2017; Wu et al., 2017). Tao et al. (2017) conducted an experiment using pulp and paper mill biosludge digestate for cultivation of *S. acuminatus*. The results showed that both micro algae can remove more than 99.99% of NH_4_-N and more than 96.9% of PO₄^−3^-P, with a biomass concentration of 2.9 g L^−1^. Moreover, *Chlorella* sp. was reported to be able to tolerate a high concentration of CO_2_ and convert it into biomass (Chiu et al., 2008). Meanwhile, Wu et al. (2017) demonstrated that *Chlorella* sp. Could grow using raw textile wastewater (Wu et al., 2017) and NH_4_^+^-N removal efficiency with aeration at 10% dilution rate and COD removal efficiency with aeration at 0% were 84% and >60%, respectively.

Industrial wastewaters are usually combined with anaerobic pretreatment or diluted suitably in order to avert the inhibition of algal growth caused by high COD concentrations since this type of wastewater contains some nutrient and high-COD as mentioned before (Wang et al., 2016). Hariz et al. (2019) used an integrated system of effluent treatment and CO_2_ fixation by *Scenedesmus* sp. UKM9 and *Chlorella* sp. UKM2. In this study*, Scenedesmus* sp. was used to treat POME in the first step. Then *Chlorella* sp. was used to treat the treated POME from the first step and capture carbon dioxide gas (CO_2_) in the second step. The results show that this system can remove 86% TN, 85% Reactive Phosphate (PO₄^−3^), and 48% COD, respectively, indicating a higher nutrient reduction in POME and greater CO_2_ fixed when compared to the individual treatment operation. Moreover, using molecular biology techniques is another way to enhance the expression of related enzymes in microalgae cells which can improve the effectiveness of wastewater treatment as well as biomass accumulation of microalgae (You et al., 2022).

As previously mentioned, heavy metals are a contaminant type present in industrial wastewater. Heavy metals are natural components of the Earth’s soil and crust that can be found in every ecosystem on Earth with a density greater than 5 g cm^−3^. Heavy metals are important for basic physiological and chemical for organisms such as plants and animals. However, some heavy metals can be poisonous to organisms. Heavy metals that are usually found in wastewater include arsenic (As), cadmium (Cd), copper (Cu), chromium (Cr), lead (Pb), manganese (Mn), Mercury (Hg), nickel (Ni), and zinc (Zn) (Ahmed et al., 2022). Microalgae show high capacities for metal biosorption. The advantages include quick uptake capability of metal, highly environmentally friendly, energy and time saving, low-cost reusable, reusable, faster growth rate compared with terrestrial plants, and nonhazardous waste generation (Kumar et al., 2015; Leong and Chang, 2020).

Heavy metals ions from wastewater can be taken up by microalgae cells through two processes, biosorption and bioaccumulation (Ahmed et al., 2022). The first mode of heavy metals uptake is referred as bioaccumulation, in which heavy metal ions are transported across the cell membrane by different systems including active and passive transport systems to be accumulated in the cells, with this process only occurring in living cells. Bioaccumulation is the slow intracellular positive diffusion and accumulation which is the mode by which heavy metals can pass to the cell of microalgae and across the cell membrane through the cell metabolic cycle. The major limitation of the process is the containment of organic carbon sources in nutritive medium for growth of the microorganism (Kumar et al., 2015; Jais et al., 2017; Kanamarlapudi et al., 2018).

The process of biosorption or rapid passive adsorption is called the independent metabolic process and occurs in living or dead cells. Heavy metal ions are attached in the cellular structure and absorbed onto the binding sites present in the cellular structure. The heavy metal ions are trapped and attached to the functional groups on the cell surface as a result of ion exchange, complexation, chelation, and micro-precipitation. The metal ions are trapped to the functional groups on the cell surface due to ion exchange, complexation, chelation, and micro-precipitation (Salam, 2019). This process is called the independent metabolic process and takes place in live or dead cells. Through ion exchange, the metal ions in the surrounding wastewater are exchanged with element ions held on the cell surface such as Ca, K and Na (Jais et al., 2017). During the process of passive biosorption, metal ions in the cationic form are physically adsorbed over the microalgal cell surface that contains functional groups like amino (-NH_2_), carboxyl (-COOH), hydroxyl (-OH), and sulfhydryl (-SH) (Ayele and Godeto, 2021). Several factors affect the biosorption process, including biomass concentration, initial metal concentration, and operational factors such as pH, temperature, concentration of the biosorbent, and concentration of the initial metal ion (Ayele and Godeto, 2021). However, the use of freely suspended microalgae as biosorbent has some limitations such as their small size, low mechanical strength, and the difficulty to separate the biomass and effluent (Salam, 2019).

## 5 Microalgae to couple wastewater treatment with microalgae for coupling CO_2_ fixation

According to the Global Energy Review 2021 by the International Energy Agency (IEA), global energy CO_2_ emissions are projected to rebound and increase by 4.8%, approaching the 2019 peak as fossil fuel demand rebounds (IEA, 2021). Global CO_2_ emissions is a critical global issue which is a primary driver of global warming through increased concentrations of greenhouse gases. Microalgae-mediated CO_2_ sequestration has been the subject of numerous research projects and has become one of the most promising strategies to reduce CO_2_ emissions. Microalgae can recycle CO_2_ into bioenergy through photosynthesis. Interestingly, CO_2_ sequestration by microalgae is an environmentally friendly and sustainable method (Brilman et al., 2013). Photosynthetic CO_2_ assimilation in microalgae consists of light-dependent and light-independent reactions. Through photosynthesis, microalgae can absorb CO_2_ emitted from various sources which is then converted into biomass and precursors of carbohydrates. These carbohydrates are then used to synthesize different biomolecules including lipids, proteins, and nucleic acids (Zeng et al., 2011; Zhang and Liu, 2021). A previous report stated that the cost of manufacturing *Chlorella* biomass is $4.87 kg^−1^, with an energy consumption of 0.96 kWh kg^−1^ of biomass (Valdovinos-García et al., 2020). Furthermore, 4,000 m^3^ of algae growth ponds might sequester up to 2.2 k tones of CO_2_ per year under natural daylight conditions (Stewart and Hessami, 2005). Another study found that 50 MW power plants might emit about 414 k tones of CO_2_ year^−1^, while a 1000-ha open raceway pond could absorb about 250 k tones of CO_2_ year^−1^. According to this study, algae might reduce CO_2_ emissions by 50% (Stepan et al., 2002).

### 5.1 Advantages of using microalgae for CO_2_ fixation

Compared to other photosynthetic organisms, microalgae have a large number of advantages for atmospheric CO_2_ capture. Microalgae cultivation systems can be tightly controlled and optimized, while its byproducts can be used to generate high value-added products due to their small size which allows them to be controlled in a closed system. They also have a higher growth rate and CO_2_ fixation capacity which results in a very high energy conservation efficiency (Paul et al., 2021). Based on their simple cell structure and high volume-to-area ratio, microalgae have a greater growth rate and CO_2_ fixation capacity than terrestrial plants. It has been reported that microalgae can grow 100 times faster and are able to fix CO_2_ while capturing solar energy with an efficiency that is 10–50 times greater than that of terrestrial plants (Singh and Ahluwalia, 2013; Zhang and Liu, 2021). Tsai et al. (2017) measured the maximum photosynthetic CO_2_ uptake rates in natural algae species, which had microalgae and green algae as the dominant species cultivated in two high-rate ponds (HRP). The maximum CO_2_ uptake rates of reactor 1 and reactor 2 were 36,299 mg m^−2^ d^−1^ and 48,829 mg m^−2^ d^−1^, respectively. Moreover, they found that the CO_2_ uptake rate of algae in reactor 1 was potentially higher than those of terrestrial plants which were calculated from the literature data. e.g., oak afforestation in Denmark, 31 years (2,371 mg m^−2^ d^−1^), Norway spruce afforestation in Germany and Italy, 93–112 years (2,763 mg m^−2^ d^−1^), and white pine afforestation in Rhode Island, United States, 115 years (2,110 mg m^−2^ d^−1^).

Another point of interest for CO_2_ sequestration using microalgae is their powerful environmental flexibility since they can tolerate and adapt to various extreme environmental conditions (Moreira and Pires, 2016; Onyeaka et al., 2021). Consequently, microalgae can play an important role in removing contaminants from wastewater from industries and agricultural activities that simultaneously produce high value-added products resulting in economic feasibility (Razzak et al., 2017; Paul et al., 2021). For instance, Yang et al. (2018) stably cultivated *Monoraphidium dybowskii* LB50 under semi-continuous culture with open raceway ponds in a desert area for 3 years, which corresponded to 30% of lipid content, 18,000 mg m^−2^ d^−1^ of biomass productivity, and a 33,000 mg m^−2^ d^−1^ CO_2_ fixation rate.

### 5.2 Development and current CO_2_ sequestration using microalgae

During the 1970’s, the U.S. Department of Energy (DOE) began research on microalgal wastewater treatment, and the recovered microalgal biomass was used for methane production. Next, a program named the “Aquatic Species Program” (ASP) funded by the U.S. Department of Energy’s Office of Fuels Development (DOE-OFD) was initiated to evaluate the potential of biodiesel production from high lipid-content algae grown in ponds, utilizing waste CO_2_ from coal fired power plants (Sheehan et al., 1998). This program achieved two major successes, including the institution of a microalgae culture collection center and a pilot-scale microalgae cultivation of two raceway ponds in New Mexico. During the 1990s, a research and development program in Japan costing over $250 million was implemented. This project was also related to bio fixation of CO_2_ and greenhouse gas emission abatement using closed microalgae photobioreactors (PBRs). However, it was later discontinued owing to the high costs associated with the reactors. In addition, the US DOE-NETL promoted microalgae research and development using closed PBRs. Moreover, other international participants consisting of Arizona Public Services, ENEL ProduzioneRicerca, EniTecnologie, ExxonMobil, and Rio Tinto have also participated in microalgae-based CO_2_ mitigation research (Bhola et al., 2014; Prasad et al., 2021). Furthermore, several other microalgae-based CO_2_ sequestration research projects are operating worldwide. The projects are mainly based on reducing the operational costs of carbon sequestration by using waste resources for algae biomass and a variety of value-added bioproducts production. Table 4 illustrates microalgae-based CO_2_ fixation research under various cultivation systems. However, most research and developmental activities on CO_2_ sequestration using microalgae is currently at the laboratory phase. Techniques to enhance microalgae cultivation for pilot-scale carbon capture are required which should focus on achieving higher biomass productivities, culture stability over long periods of time, economical harvesting methods, and improved biomass-to-fuels transformation technologies (Prasad et al., 2021).

**TABLE 4:** CO_2_ fixation using various microalgae species under various cultivation systems.

Microalgae	Cultivation system	Culture medium	CO_2_ concentration	CO_2_ fixation rate (g L^−1^/d^−1^)	References
*Chlorella* sp	Illuminated incubator chamber	‒	15%	0.097	Kassim and Meng, (2017)
Algae consortium^a^	High-rate pond (HRP)	Natural water	‒	0.159	Tsai et al. (2017)
*Chlorella* sp. UKM2	Glass culture bottles	Palm oil mill effluent (POME)	10%	0.829	Hariz et al. (2019)
*Chlorella* sp	Bubble column photobioreactors (BCR)	Domestic wastewater puls poultry waste	Flue gas containing 10% CO_2_	0.261	Yadav et al. (2020)
*C. vulgaris*	Bubble column photobioreactors (BCR)	BG-11 medium	7%	0.633	Barahoei et al. (2020)
*C. vulgaris*	Open raceway pond	BG-11 medium	5%	0.290	Yu et al. (2020)
*C. sorokiniana* GS03, *Heynigia riparia* SX01	Bubble column PBRs	BG-11 medium	5%15%	0.660, 0.710	Jin et al. (2021)
*Botryococcus braunii Scenedesmus* sp	Glass culture bottles	BG-11 medium	20% mixed with N_2_	0.532, 2.177	Rodas-Zuluaga et al. (2020)
*S. almeriensis*	Vertical bubble column photo-bioreactor (VBC-PBR)	Modified Mann and Myers medium	3% mixed with N_2_ and O_2_	0.240	Molino et al. (2019)

a Microalgae and green algae as the dominant species.

## 6 Challenges and future perspective

Microalgae are a third-generation feedstock and a promising starting material for bio-renewables production that can compensate for fossil fuel and the first generation of biofuel that comes from corn, soy, and sugar cane. Nowadays, the combination of microalgae and clean technology such as biorefineries and biofuel production can release almost zero waste into the environment (Wang et al., 2022). Although CO_2_ sequestration using microalgae has an environmental benefit, it is limited by the high expense of CO_2_ capture and transport, as well as significant CO_2_ losses during microalgae culture. This constraint may result in high manufacturing costs. The normal compression process flow through the pipeline requires a lot of energy and raises transport expenses. Economic analyses revealed that combining the supplementation of the two carbon sources significantly reduced the cost of carbon procurement, dropping from $1.37 kg^−1^ when using 1% (v/v) CO_2_ alone as the carbon source to $0.86 kg^−1^ when using 1% (v/v) CO_2_ together with NaHCO_3_ (0.5 g L^−1^) while increasing the yield of FAME by nearly 80%. These results suggest that mixing CO_2_ with NaHCO_3_ is a more cost-effective way to supply carbon to microalgae for biodiesel production (Nayak et al., 2018). In addition, there are some gaps that must be considered as an environmental point for the further progress of larger-scale production. For instance, ammonia (NH_3_) is an inorganic fertilizer that is used to attain an appropriate algal biomass growth rate and productivity. For every 1 kg of NH_3_ generated, 1.2 kg of CO_2_ is released, resulting in environmental deterioration and the discharge of chemical reagents into the environment during the process of microalgae production. This issue must be considered as an important point to manage the whole microalgal production factory with clean energy to achieve environmental sustainability (Wang et al., 2022). The life cycle assessment (LCA) can be used to evaluate the environmental effects and energy concerns, which is an appropriate choice for future improvements in the environmental field (De Souza et al., 2019). According to a previous study, LCAs can be used to show potential ways to prove that applying microalgae contributes to positive feedback to the environment (Peter et al., 2021).

With regard to the economic concerns, using a microalgae system to remove nitrogen, phosphorus, and dissolved organic carbon from several types of wastewaters is far more sustainable than conventional systems because a microalgae system can operate outside in sunlight which serves to reduce costs (Mohsenpour et al., 2021). However, due to the open nature of open systems, additional bacteria may contaminate the culture. Contamination with other microorganisms could be a limitation since this process is conducted in non-sterile conditions, leaving only a few strains that are sufficiently resistant, fast growing, and tolerant of extreme conditions that can be grown in open reactors. A potential solution to this problem is using co-culture between microalgae and other microorganisms i.e., bacteria, yeast, and fungi to accelerate wastewater treatment (Mohsenpour et al., 2021). Consequently, the role of co-culture requires further exploration in wastewater scales with synthetic and real wastewater (Fazal et al., 2018). Further studies should investigate the interaction between microalgae and other microorganisms and study gene and transcription factors involved in the mechanism of microorganisms in wastewater. Omics approaches using targeted genome editing such as clustered regularly interspaced short palindromic repeats (CRISPR) to develop an effective solution to improve the future of microalgae for value added bioproducts in more commercial and potential is highly recommended (Kumar et al., 2021).

Another important challenge is that the composition of nutrients included in various municipal wastewaters should be taken into account when treating microalgae. The ratio and concentration of nutrients should be balanced by mixing different types of wastewaters in order to meet wastewater discharge guidelines (Ahmad et al., 2022). Besides the cost of algal biomass generation, post-cultivation biomass recovery can account for up to 30% of the total production expenses. Microalgae cells are tiny (2–20 µm) and have a density similar to water, which is one of the primary reasons for this. There are many challenges in large-scale agriculture, including efficient biomass recovery. Due to the large amount of energy that is needed, another problem that arises is the removal of microalgae from wastewater produced by the many different industrial wastewater treatment procedures (Amenorfenyo et al., 2019). Recent research indicates that the concentration of microalgal biomass will require a two-stage process: the utilization of flocculation in combination with sedimentation. Bio-flocculation is the better choice between these two methods because it cuts down on the costs of chemicals while keeping the efficiency of the biomass (Barros et al., 2015; Alam and Wang 2019). However, there are very limited research focusing on economic evaluation. The economical practicability of the process in real wastewater treatment under operation are still required for further study.

Additional carbon sequestration research on microalgae should be focused on the factors that influence the growth of microalgae, such as their wide range of tolerance and sensitivity to temperature, pH, irradiance and nutritional conditions, where even minor changes in cultivation conditions can have a significant impact on product yields. Additionally, carbon sequestration research on microalgae should focus on aspects affecting their growth, such as their wide range of tolerance and sensitivity to temperature, pH, irradiance, and nutritional circumstances, where even minor changes in production conditions affect product output (Molazadeh et al., 2019). Using modeling to assist the optimization of in the further studies is an alternative choice. Finally, the durability and economic viability of large-scale microalgae carbon sequestration are contingent upon a thorough knowledge of the photosynthetic process, the improvement of growth factors, and the development of technical infrastructure.

## 7 Conclusion

In summary, microalgae are photosynthetic microorganisms that play a vital role in the bioremediation of several types of wastewaters, including removal of N, P, and C, the reduction of BOD, as well as heavy metal removal. The integration of microalgae into several types of wastewaters can decrease the cost of wastewater treatment, obtain a lower footprint in terms of energy consumption, and provide environmental sustainability compared to existing conventional wastewater treatment processes. It is highly vital to evaluate the environmental effects of large-scale use of microalgae bioenergy if it is to be developed into an alternative energy to reduce fossil fuel consumption. Moreover, integrated microalgal biorefinery not only solves environmental problems, but also acts as a producer which can produce high added-value bio compounds such as biofuel, biodiesel, and other valuable compounds.
