# Corrigendum: Microalgae-based wastewater treatment for developing economic and environmental sustainability: Current status and future prospects

**DOI:** 10.3389/fbioe.2022.1048819

**Published:** 2022-10-21

**Authors:** Piroonporn Srimongkol, Papassara Sangtanoo, Pajareeya Songserm, Wannapawn Watsuntorn, Aphichart Karnchanatat

**Affiliations:** ^1^ Center of Excellence in Bioconversion and Bioseparation for Platform Chemical Production, Institute of Biotechnology and Genetic Engineering, Chulalongkorn University, Bangkok, Thailand; ^2^ Panyapiwat Institute of Management Demonstration School, Nonthaburi, Thailand

**Keywords:** microalgae, wastewater treatment, biomolecule production, biorefineries, bioenergy

## Abstract

**Figure:**
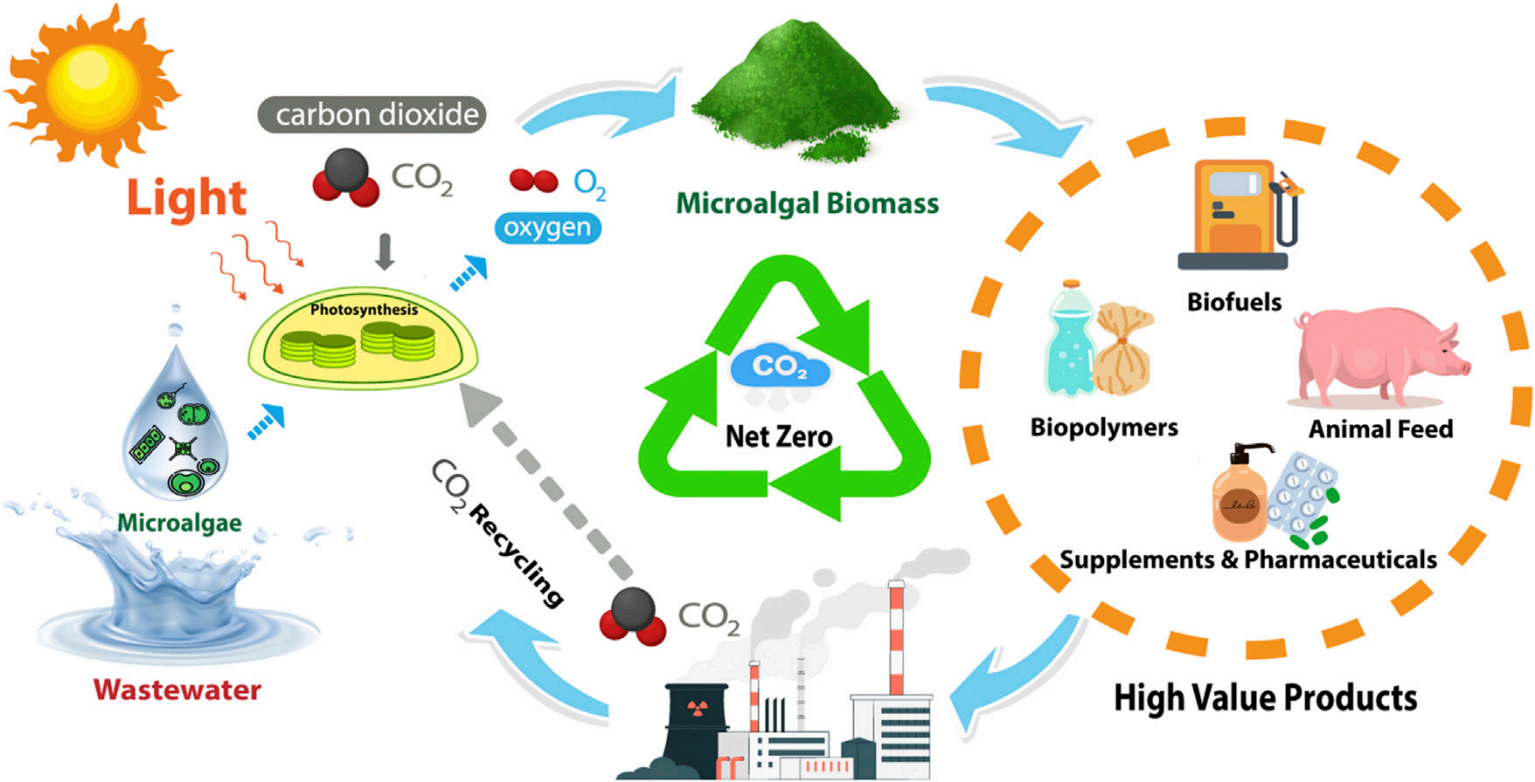
A biorefinery based on microalgae promotes a circular-green economic model.

A biorefinery based on microalgae promotes a circular-green economic model.

In the published article, there was an error in the **Graphical abstract** as published. Some of the words were not deleted**.** The corrected **Graphical abstract** and its caption, “A biorefinery based on microalgae promotes a circular-green economic model,” appear above.

In the published article, there was an error in Figure 1 as published. Some of the words were misspelled**.** The corrected Figure 1 and its caption, “Wastewater integrated algal-biorefinery for biofuel and other value-added compound productions (created with BioRender.com),” appear below.

**FIGURE 1:**
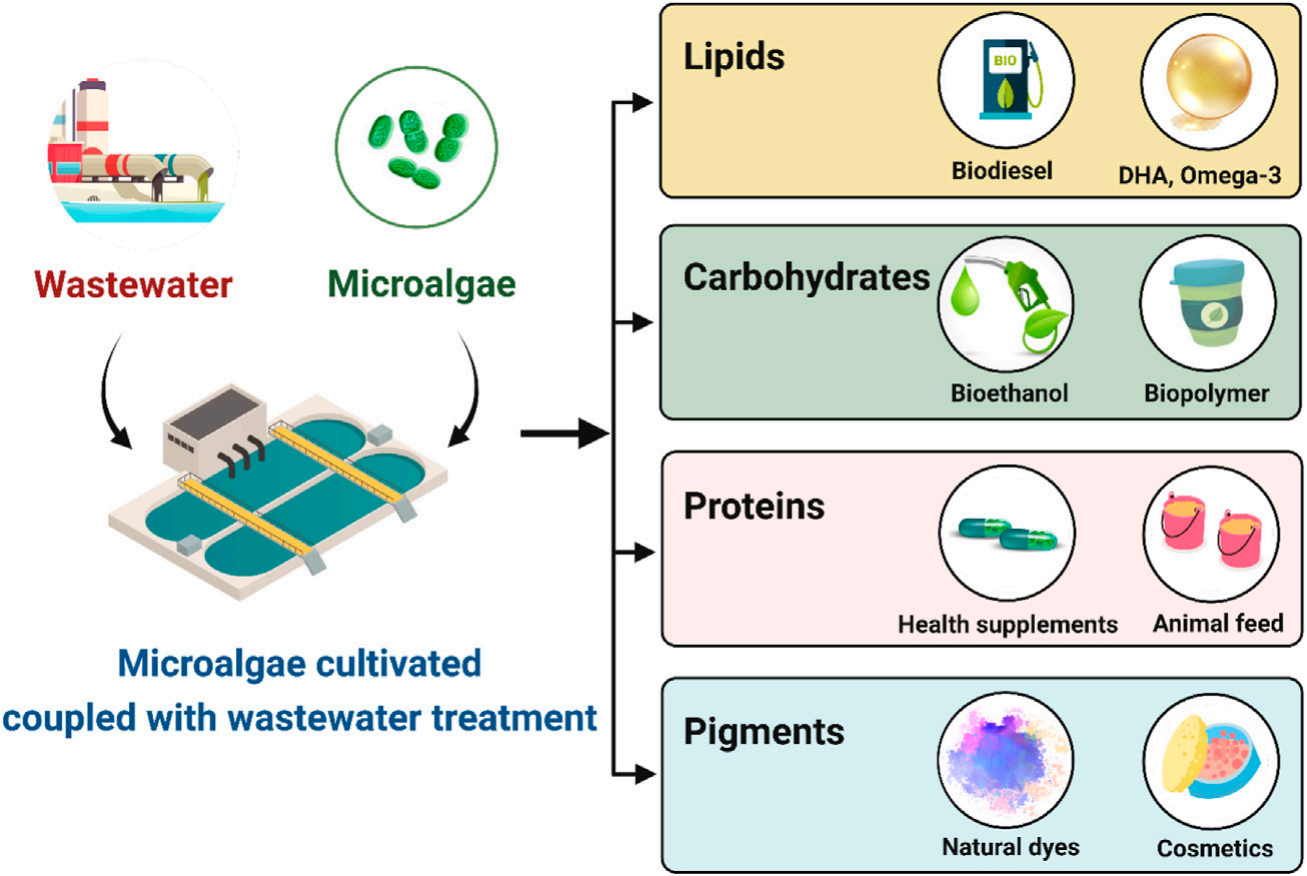
Wastewater integrated algal-biorefinery for biofuel and other value-added compound productions (created with BioRender.com).

The authors apologize for this error and state that this does not change the scientific conclusions of the article in any way. The original article has been updated.

